# Subpleural injection of gelatin sponge particles to reduce pneumothorax incidence in CT-guided lung biopsies: a retrospective single-center case-control study

**DOI:** 10.1186/s12885-025-13911-9

**Published:** 2025-03-23

**Authors:** Zi-Yi Zhu, Ya-Qin Yun, Hao Li, Zhong-Qiang Qin, Zhen Qian, Yun Zhu, Shu-Hua Li, Bo Xie, Mu Yuan

**Affiliations:** 1 Department of Interventional Radiology, The First Affiliated Hospital of Bengbu Medical University, No.287 Changhuai Road, Bengshan District, Bengbu, 233004 Anhui Province China; 2 Department of Radiology, The First Affiliated Hospital of Bengbu Medical University, No.287 Changhuai Road, Bengshan District, Bengbu, 233004 Anhui Province China

**Keywords:** CT-guided lung biopsy, Pneumothorax, Gelatin sponge particle, Subpleural injection

## Abstract

**Background:**

To evaluate the efficacy and safety of track sealing using subpleural injection of gelatin sponge particles in reducing the incidence of pneumothorax after percutaneous CT-guided lung biopsy.

**Methods:**

This study conducted a retrospective analysis of 1,026 patients who underwent CT-guided lung biopsy at our center from January 2022 to July 2024. After propensity score matching (PSM) to minimize the impact of confounding variables like smoke, lesion diameter, and tract length, 338 patients were ultimately included and assigned to the sealant group (169 patients) or the non-sealing group (169 patients) according to whether using the gelatin sponge particles sealing after needle withdraw to the subpleural area. Clinical and operative characteristics data were collated from electronic medical records (EMR) and Picture Archiving and Communication Systems (PACS). A multivariable logistic regression analysis was conducted to identify predictors of pneumothorax.

**Results:**

In the sealing group, the incidence of pneumothorax was 14.8%, whereas it was significantly higher in the non-sealing group at 23.7% (*p* < 0.05). There was no significant difference in the chest tube placement rates of 3% and 1.8% (*p* = 0.723). Importantly, no significant complications, such as air embolism, were observed in either group. A multivariate logistic regression analysis, using a propensity score-matched cohort, identified patient emphysema (OR = 2.35 [1.22–4.51], *p* = 0.01) and the tract length (OR = 1.25 [1.01–1.55], *p* = 0.042) as significant risk factors for pneumothorax. Furthermore, gelatin sponge particle needle-tract sealing demonstrated a marked and statistically significant reduction in the risk of pneumothorax (OR = 0.5 [0.27–0.91], *p* = 0.024), highlighting the distinct advantages and clinical value of this treatment in preventing such complications.

**Conclusions:**

The gelatin sponge particle subpleural sealing technique can effectively reduce the incidence of pneumothorax in patients undergoing percutaneous CT-guided lung biopsy.

## Introduction

Percutaneous trans-thoracic lung biopsy (PTLB) has become a pivotal technique for obtaining lung tissue samples, presenting a practical alternative to other methods. However, a significant challenge to this procedure is the frequent occurrence of postoperative pneumothorax, affecting between 8.6% and 45% of patients [1–3]. As a result, clinicians have been striving to minimize the occurrence of pneumothorax. Previous studies have revealed that several independent prognostic factors, such as the diameter of the trocar [4, 5], contact with the pleura [6, 7], and prone or lateral body position [8, 9], influence the likelihood of pneumothorax. Despite this understanding and ongoing efforts, the rate of pneumothorax remains elevated. Considering these factors, introducing sealing materials after trocar insertion has been suggested as a proactive approach that could decrease the incidence of pneumothorax.

Using liquid sealants, such as saline [10], or solid sealants, like autologous blood clots or vessel plugs [11], effectively diminishes the occurrence of pneumothorax. Reports indicate that the incidence of pneumothorax ranges between 6.9% and 29% [10, 12, 13]. However, these embolization methods are not widely adopted due to limited data on the rate of delay pneumothorax and the complexities and expenses involved in their preparation. Therefore, a pressing demand exists for an embolization material that offers ease of preparation, exceptional safety, inexpensive, and a well-defined efficacy profile in clinical settings.

The gelatin sponge is a cost-efficient and easy-to-prepare material, offering exceptional absorbency and expandability. Previous research has indicated that applying a gelatin sponge for track sealing can considerably diminish the likelihood of pneumothorax [14, 15]. Nevertheless, there is variability in the methodology’s specifics, particularly regarding the most suitable injection site and the ideal particle size. Prior investigations have demonstrated that the subpleural administration of gelatin sponge slurry (approximately 5-mm sponge cubes mixed with 3 ml saline) as a sealant can notably decrease the incidence of pneumothorax compared to saline [16]. However, no research has been conducted to examine whether the utilization of gelatin sponge particles, which present more homogeneity in size and shape, as a sealant for subpleural injection yields comparable efficacy and safety.

Therefore, our study aimed to evaluate the efficacy of the subpleural injection of gelatin sponge particles as a sealant after PTLB to reduce the rate of pneumothorax. In addition, PSM (1:1) was used to reduce the influence of data bias and confounding factors, as track sealing was more likely to be used in patients with a higher risk of pneumothorax.

## Materials and methods

### Study design

**Fig. 1:**
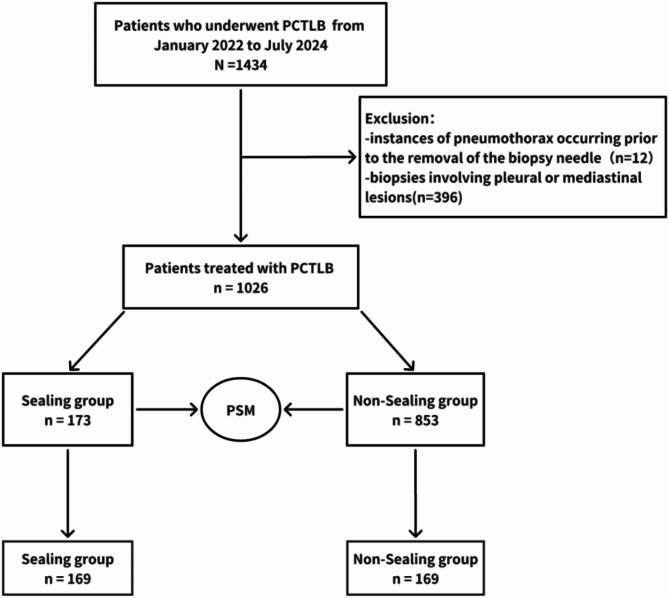
The flowchart of this study

To ensure the scientific rigor and comprehensive nature of the analysis, this study was subject to approval by the Ethics Review Committee, with the requirement for patient informed consent being waived. The study encompassed all patients who underwent percutaneous CT-guided PTLB between January 2022 and July 2024, resulting in 1,026 cases. During the data screening stage, specific cases were excluded, namely: (1) instances of pneumothorax occurring before the removal of the biopsy needle; (2) biopsies involving pleural or mediastinal lesions. To reduce the potential confounding factors, including emphysema, lesion diameter, and tract length, propensity score matching was employed to adjust the dataset, resulting in a refined sample of 338 patients. The cohort was divided into the sealing group, comprising 169 patients who underwent gelatin sponge particles for track sealing, and the non-sealing group (169 patients). All patients were provided with comprehensive information regarding the potential risks and complications, including pneumothorax and bleeding, and subsequently provided informed consent. Furthermore, patients were informed that using gelatin sponge particles could reduce the risk of pneumothorax (Fig. 1).

### Percutaneous lung biopsy and track sealing

**Fig. 2:**
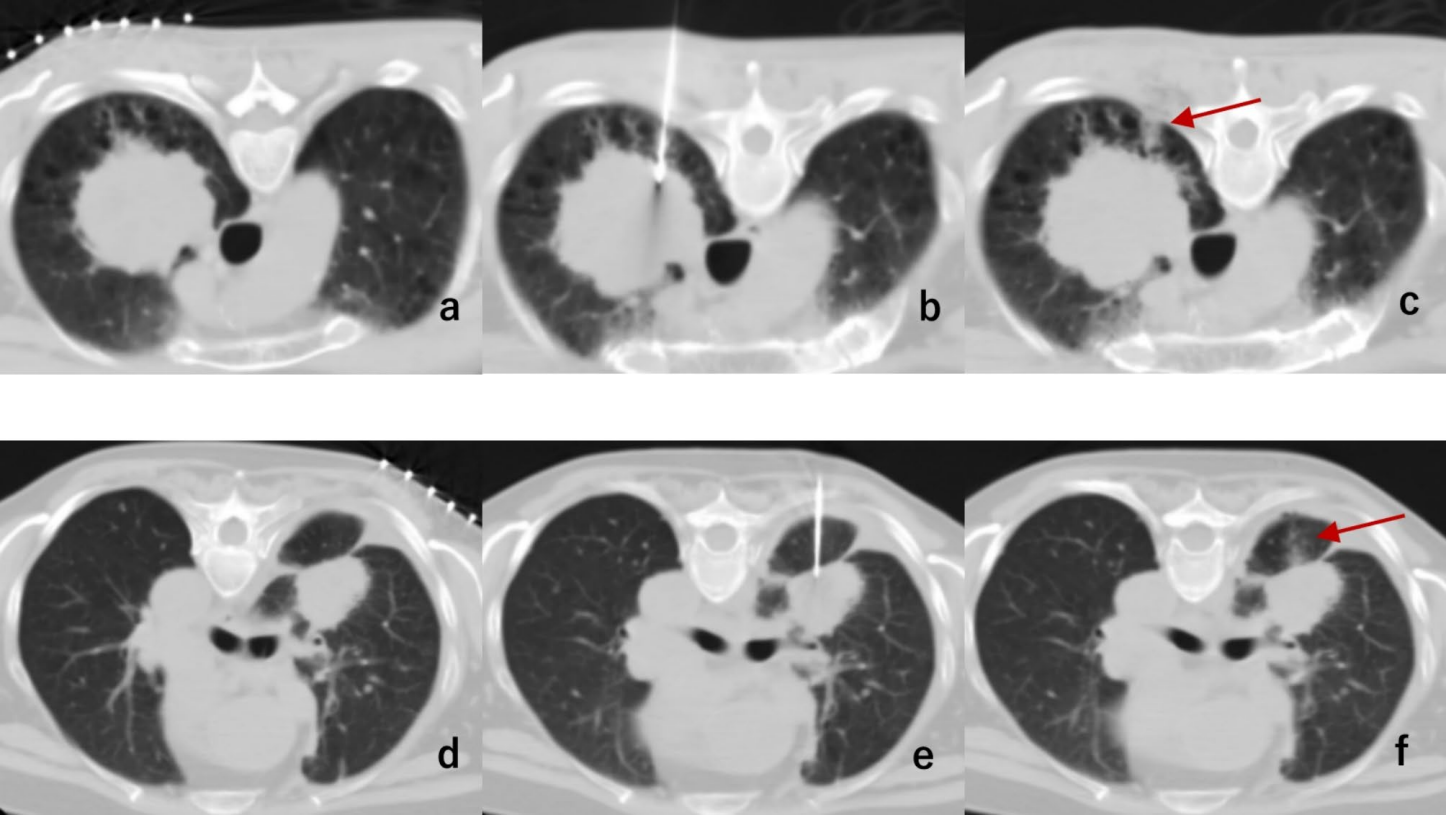
Axial computer tomography (CT) fluoroscopic image of 2 lung biopsy procedures with tract sealing. A 59 (Y/M) patient with emphysema and a suspicious left upper lobe lung lesion (**a-c**), and another 67(Y/F) patient with a right upper lobe lung lesion contact with fissure (**d-f**). Image showing the pulmonary bullae surrounding the lesion (**a**), the trocar needle incidentally transgressed the bullae (**b**). withdraws the needle to a 5–10 mm subpleural area and injects the sponge particle, forming a well-defined opacity(arrow). No pneumothorax was detected after the needle was removed (**c**). Image showing the lesion contact with fissure (**d**), the trocar needle passed the fissure (**e**), tract sealing was performed, and no pneumothorax was detected after the needle was removed (**f**)

A computed tomography scanner guided all procedures, and a percutaneous biopsy was performed by one of five interventional physicians, each with more than five years of experience (Z.Z, Q.Q, Z.Q, B.X, M.Y.). Before the biopsy, the exact location of the lesion was determined using enhanced CT and real-time lung scanning, carefully avoiding vital areas such as bone and pulmonary bullae. The patient’s prone, supine or lateral decubitus position was chosen to ensure the shortest puncture path to the lesion. Patients were instructed to breathe freely during the procedure, and a precise puncture trajectory was mapped out. A 17-G trocar needle (Argon Medical, Frisco, TX, America) was used to perform the biopsy along this predetermined optimal path. Approximately 5-10 ml of 1% lidocaine was administered for local anesthesia, and the needle gradually inserted the lesion along the predetermined trajectory. An 18-G cutting biopsy needle (Argon Medical, Frisco, TX, America) was passed coaxially, and 2–3 sample strips were obtained (Fig. 2).

After the biopsy, the needle tip was retracted 5–10 mm below the pleura. The cutting needle was removed, and the sealant was injected until the sheath of the biopsy needle was removed from the skin. As for lesions requiring a fissure pass, our standard procedure involves withdrawing the trocar 5–10 mm beneath the fissure and evenly distributing the gelatin sponge particle: half to seal the fissure passage and the remaining half to seal the pleural breach. The steps to prepare the sealant are as follows. Gelatin sponge particles (700–1000 μm, approximately 100 mg; Alicon Medical Company, Hangzhou, China) are placed in a 10 ml syringe with a three-way stopcock, and the excess air is evacuated. Inject 3 ml saline into the 10 ml syringe loaded with sponge particles through a three-way stopcock. Mix thoroughly and aspirate repeatedly until a thick suspension is obtained, ensuring no air bubbles. The suspension concentration should be high to reduce the likelihood of vessel embolization.

After the biopsy, a chest CT scan was performed to check for postoperative complications at the end of the biopsy, such as pneumothorax, pulmonary hemorrhage, or air embolism. It was assessed and managed according to the Society of Interventional Radiology standards [17]. Patients exhibiting symptomatic (dyspnea, hypoxemia) and progressive pneumothorax (in the subsequent CT scan images captured at intervals of 3 to 5 min), or those whose pneumothorax encompassed over 30% of the hemithorax, according to the most recent CT image at the end of the biopsy, were deemed suitable for chest drainage. At our facility, the radiologist swiftly performs chest drainage in the CT lab using an 8.5 F pigtail catheter. Short-term aspiration is carried out based on the pneumothorax’s progression until the compressed lung expands or dyspnea is alleviated. Afterward, the catheter is linked to a water-sealed bottle, functioning as a one-bottle underwater seal chest drainage system for persistent trans-thoracic air drainage. The patient is then sent back to the referring department for a 24-hour observation. Notably, the precise drainage volume wasn’t documented as the main emphasis was on lung expansion or dyspnea relief. During this period, patients are advised to rest in bed in a supine position and avoid coughing. Vital signs, including blood pressure, heart rate, and oxygen saturation, are closely monitored for signs of delayed pneumothorax, like breathing difficulties or oxygen desaturation. In case of such symptoms, an X-ray scan is performed, and a thoracic surgeon is consulted to assess the need for surgical intervention. The drainage tube is removed prior to discharge or at a local hospital once a chest CT or X-ray confirms the resolution of the pneumothorax.

### Data collection

The clinical features of patients are comprehensively recorded through the electronic medical record (EMR) system, including sex, age, hypertension, diabetes, and smoking history. Preoperative CT images in the Picture Archiving and Communication System (PACS) are carefully reviewed, focusing on the presence of emphysema and pulmonary bullae. The diagnosis of emphysema was primarily based on preoperative chest CT scans, which were reviewed by two radiologists (Y.Z, S.L.) with more than five years of experience and categorized as either present or absent emphysema. Due to the limited number of cases, we decided not to subdivide the cases according to the specific type and severity of emphysema. Instead, we grouped Centrilobular, panlobular, and paraseptal emphysema under the umbrella term “emphysema” [18]. The characteristics of the lesions are accurately documented, including lesion location (lung lobe) and diameter. Procedure-related information is recorded in detail, such as patient positioning, track length, and whether a fissure was passed. The diameter was defined as the maximum length of the lesion, while tract length referred to the distance from the pleura to the lesion along the needle trajectory. Fissure passes, referred to as the fissure, were transgressed by the coaxial needle. Complications are monitored, including pulmonary hemorrhage, pneumothorax, air embolism, and whether chest tube placement was required. Pneumothorax was defined as the presence of air in the pleural space between the parietal and visceral pleura. Two senior interventional and radiology physicians meticulously record and review all data to ensure accuracy and completeness (Z.Z, M.Y.).

### Statistical analysis

We used the statistical software R (provided by The R Foundation at http://www.R-project.org) and FreeStatistics version 1.2 for all analyses. Statistical significance was determined using a two-tailed p-value of less than 0.05. Categorical variables were presented as proportions (%), while continuous data were appropriately presented as mean ± standard deviation (SD) or median with interquartile range (IQR). We implemented propensity score matching (PSM) to reduce potential bias using a 1:1 nearest neighbor matching algorithm with a caliper width 0.2. Variables included in the propensity score were sex, age, diabetes status, hypertension, smoking history, emphysema, diameter, distance, position, lobe, and fissure. Multivariable Cox regression analyses were performed to evaluate the independent association between prognostic factors and the rate of pneumothorax.

## Result

### Population

After propensity score matching (PSM), 338 patients were included in the study, with 169 in the sealing group and 169 in the non-sealing group (Fig. 1). After matching, there were no significant differences between the two groups in baseline characteristics such as sex, age, diabetes, hypertension, smoking history, emphysema, lesion diameter, tract length, patient position, lobe, contact with fissure, lesion type, fissure pass, and chest tube (*P* > 0.05) (Table 1).

**Table 1:** Characteristics of sealing and non-sealing group before and after propensity score matching

	Before matching			After matching		
	Non-sealing group(*n* = 853)	Sealing group (*n* = 173)	*P*	Non-sealing group(*n* = 169)	Sealing group (*n* = 169)	*P*
Sex, n (%)			0.352			0.569
Female	343 (40.2)	63 (36.4)		57 (33.7)	62 (36.7)	
Male	510 (59.8)	110 (63.6)		112 (66.3)	107 (63.3)	
Age, Mean ± SD	66.3 ± 10.1	67.0 ± 11.6	0.396	67.3 ± 9.7	66.9 ± 11.7	0.746
Diabetes, n (%)			0.707			0.877
No	743 (87.1)	148 (86)		144 (85.2)	145 (85.8)	
Yes	110 (12.9)	24 (14)		25 (14.8)	24 (14.2)	
Hypertension, n (%)			0.266			0.645
No	608 (71.3)	116 (67.1)		110 (65.1)	114 (67.5)	
Yes	245 (28.7)	57 (32.9)		59 (34.9)	55 (32.5)	
Smoke, n (%)			0.023			0.359
No	704 (82.5)	130 (75.1)		135 (79.9)	128 (75.7)	
Yes	149 (17.5)	43 (24.9)		34 (20.1)	41 (24.3)	
Emphysema, n (%)			0.853			0.649
No	536 (62.8)	110 (63.6)		111 (65.7)	107 (63.3)	
Yes	317 (37.2)	63 (36.4)		58 (34.3)	62 (36.7)	
Diameter, Mean ± SD	3.5 ± 1.7	3.3 ± 1.8	0.136	3.1 ± 1.5	3.2 ± 1.8	0.408
Tract length, Mean ± SD	2.3 ± 1.3	2.7 ± 1.4	< 0.001	2.5 ± 1.4	2.7 ± 1.3	0.384
Position, n (%)			0.794			0.883
Supine	334 (39.2)	70 (40.5)		67 (39.6)	68 (40.2)	
Prone	408 (47.8)	82 (47.4)		76 (45)	80 (47.3)	
Left lateral	67 (7.9)	15 (8.7)		19 (11.2)	15 (8.9)	
Right lateral	44 (5.2)	6 (3.5)		7 (4.1)	6 (3.6)	
Lobe, n (%)			0.52			0.91
Left upper lobe	261 (30.6)	45 (26)		41 (24.3)	44 (26)	
Left lower lobe	108 (12.7)	25 (14.5)		22 (13)	25 (14.8)	
Right upper lobe	232 (27.2)	51 (29.5)		57 (33.7)	51 (30.2)	
Middle lobe	93 (10.9)	24 (13.9)		26 (15.4)	23 (13.6)	
Right lower lobe	159 (18.6)	28 (16.2)		23 (13.6)	26 (15.4)	
Contact with fissure, n (%)			0.965			0.702
No	775 (90.9)	157 (90.8)		155 (91.7)	153 (90.5)	
Yes	78 (9.1)	16 (9.2)		14 (8.3)	16 (9.5)	
Lesion type, n (%)			0.909			0.47
Solid	739 (86.6)	151 (87.3)		139 (82.2)	147 (87)	
Ground glass	52 (6.1)	11 (6.4)		14 (8.3)	11 (6.5)	
Cavity	62 (7.3)	11 (6.4)		16 (9.5)	11 (6.5)	
Fissure pass, n (%)			0.214			0.685
1	815 (95.7)	169 (97.7)		167 (98.8)	165 (97.6)	
>1	37 (4.3)	4 (2.3)		2 (1.2)	4 (2.4)	

### Pneumothorax incidence and chest tube insertion rate in two groups of PTLB patients

After propensity score matching analysis, we found that the pneumothorax incidence was significantly lower in the gelatin sponge particle sealing group compared to the control group (14.8% vs. 23.7%, *P* = 0.038). The difference in the proportion of chest tube insertions was insignificant (3.0% vs. 1.8%, *P* = 0.723) (Table 2). No severe complications, such as air embolism, were observed in either group.

**Table 2:** The incidence of pneumothorax and the rate of chest tube insertion in two groups of patients

Complication	Sealing group (*n* = 169)	Non-sealing group (*n* = 169)	*P*-Value
Pneumothorax	25 (14.8%)	40 (23.7%)	0.038
Chest tube insertion	5 (3.0%)	3 (1.8%)	0.723

### Univariate and multivariate analysis of pneumothorax in the sealing group

In the univariate analysis, track sealing was significantly associated with a reduced incidence of pneumothorax (OR = 0.56 [0.32–0.97], *p* = 0.04). Additionally, sex (OR = 1.86 [1.00-3.43], *p* = 0.049), older age (OR = 1.03 [1.00-1.06], *p* = 0.025), and the presence of significant emphysema (OR = 2.58 [1.49–4.48], *p* = 0.001) were associated with an increased incidence of pneumothorax. Furthermore, the left lateral decubitus position (OR = 2.96 [1.27–6.88], *p* = 0.012) and the tract length (OR = 1.26 [1.04–1.53], *p* = 0.018) were also identified as a risk factor for pneumothorax.

Multivariate analysis further confirmed some of the univariate findings, identifying two independent risk factors for pneumothorax: emphysema (OR = 2.35 [1.22–4.51], *P* = 0.01) and tract length (OR = 1.25 [1.01–1.55], *P* = 0.042). The analysis also clearly indicated that gelatin sponge particle sealing significantly reduced the risk of pneumothorax (OR = 0.50 [0.27–0.91], *P* = 0.024) (Table 3).

**Table 3:** Univariate and multivariate regression analyses on risk factors for pneumothorax

	Univariate analysis		Multivariate analysis	
	OR (95% CI)	*P*-value	OR (95% CI)	*P*-value
Sex	1.86(1 ~ 3.43)	0.049	1.24(0.59 ~ 2.6)	0.576
Age	1.03(1 ~ 1.06)	0.025	1.02(0.99 ~ 1.06)	0.178
Diabetes	1.09(0.51 ~ 2.32)	0.821	1.28(0.55 ~ 2.97)	0.567
Hypertension	0.77(0.43 ~ 1.4)	0.394	0.67(0.34 ~ 1.32)	0.25
Smoke	0.75(0.38 ~ 1.5)	0.422	0.58(0.26 ~ 1.25)	0.163
Emphysema	2.58(1.49 ~ 4.48)	0.001	2.35(1.22 ~ 4.51)	0.01
Diameter	1.14(0.98 ~ 1.33)	0.09	1.06(0.88 ~ 1.29)	0.53
Tract length	1.26(1.04 ~ 1.53)	0.018	1.25(1.01 ~ 1.55)	0.042
Position (vs. Supine)				
Prone	1.24(0.67 ~ 2.29)	0.494	1.16(0.52 ~ 2.55)	0.718
Left lateral	2.96(1.27 ~ 6.88)	0.012	2.96(0.99 ~ 8.87)	0.053
Right lateral	1.63(0.41 ~ 6.42)	0.486	1.92(0.42 ~ 8.77)	0.4
Lobe (vs. Left upper lobe)				
Left lower lobe	0.88(0.36 ~ 2.15)	0.782	1.04(0.34 ~ 3.18)	0.941
Right upper lobe	0.55(0.26 ~ 1.19)	0.131	0.64(0.26 ~ 1.56)	0.327
Middle lobe	0.95(0.4 ~ 2.27)	0.916	0.98(0.32 ~ 2.98)	0.975
Right lower lobe	1.49(0.66 ~ 3.34)	0.335	1.13(0.39 ~ 3.3)	0.817
Contact with fissure	1.31(0.54 ~ 3.2)	0.551	0.86(0.3 ~ 2.53)	0.789
Lesion type (vs. Solid)				
Ground glass	0.37(0.09 ~ 1.63)	0.191	0.59(0.12 ~ 2.88)	0.514
Cavity	2.15(0.92 ~ 5.04)	0.079	2.1(0.81 ~ 5.48)	0.129
Fissure pass	0.84(0.1 ~ 7.29)	0.872	1.23(0.11 ~ 13.85)	0.865
Tract sealing	0.56(0.32 ~ 0.97)	0.04	0.5(0.27 ~ 0.91)	0.024

## Discussion

This study was a retrospective analysis of 1,026 patients who underwent CT-guided PTLB at our center between January 2022 and July 2024. To minimize the impact of confounding factors such as emphysema, tumor diameter, and puncture distance, propensity score matching was applied to the data, resulting in a final sample of 169 patients. Our results showed that the sealing group had a significantly lower incidence of pneumothorax than the non-sealing group (14.8% vs. 23.7%, *P* < 0.05) without substantially increasing complication rates.

Since 1974, when McCartney et al. [19] first suggested that sealing the biopsy tract could reduce pneumothorax complications, many sealing techniques and materials have been proposed. In a systematic review and meta-analysis, Huo et al. [20] compared the results of studies using different methods to reduce the incidence of pneumothorax. They found that compared with tract plug, rapid rollover, hydrogel plug, and blood patch, saline as a tract sealant was more effective in preventing serious pneumothorax and reducing medical expenses. However, this finding should be interpreted prudently because the eligibility (e.g., tumor size, emphysema) was not identical between the studies. Therefore, Others doubt whether saline can be relied upon as a reliable sealant under some harsh conditions. Dheur et al. [16] confirmed this view in their prospective study, which showed that using gelatin sponge slurry to seal the channel was more effective in reducing pneumothorax (12.1% vs. 24.6%, *p* = 0.008) and shortening hospital stays than saline sealing (*p* = 0.003) in a cohort with a majority of emphysema (56.7% vs. 50.8, *p* = 0.33). This may be due to the saline’s rapid absorption and diffusion in lung tissue and its uneven distribution under gravity, which can lead to poor embolization and stability.

Compared to saline, gelatine sponges can absorb up to 45 times their weight in blood, effectively sealing the tract [21]. The effectiveness of gelatine sponge products has also been confirmed in a series of retrospective studies. Tran et al. [22] showed that although gelatin sponge slurry did not considerably reduce the incidence of pneumothorax, it significantly reduced the drain placement rate (10.7% vs. 6.9%, *p* = 0.01). Given the heterogeneity of the patients and trocars included, the efficacy of the gelatin sponge may have been underestimated. A subsequent retrospective study by Rémi Grange et al. [23] showed that the incidence of pneumothorax (26.1% vs. 44.9%; *p* < 0.001) and the rate of chest tube placement (2.3% vs. 4.8%; *p* < 0.001) were significantly reduced after embolization with gelatine sponge suspension. Hadrien Renier et al. [24] also confirmed that compared to the non-channel embolization group, the incidence of pneumothorax in the channel embolization group decreased from 25.8 to 10% (*p*<0.0001), and the rate of chest tube placement decreased from 12.2 to 3.5% (*p* = 0.0005). In a study investigating the incidence of early and late pneumothorax after embolization, Sum et al. [25] showed that the use of gelatin sponge slurry to seal the biopsy tract significantly reduced the rate of pneumothorax (30% vs. 42.1%, *p* = 0.032). More interestingly, the risk of immediate and delayed pneumothorax in non-peripheral lesions was significantly higher in patients without gelatin sponge slurry sealing (*P* = 0.001 and *P* = 0.002, respectively).

In recent years, gelatine sponge powder has gradually been used as a new and straightforward sealant for tract embolization [26]. Gelatin sponge powder is a non-toxic, non-antigenic, absorbable, and inexpensive hemostatic material that has long been used in various interventional procedures as a vascular occlusion material. Compared to commercially available gelatin sponge particles, gelatin sponge slurry must be prepared manually. Uneven slurry preparation may lead to outer sheath embolism, and gelatin sponge slurry’s re-preparation may prolong operation time and increase the risk of intrapulmonary hemorrhage and pneumothorax. Although no extensive prospective studies are comparing the efficacy and safety of gel sponge particles with gelatin sponge slurry, one animal study found that the rate of pneumothorax was significantly lower in the embolization group (25.0 vs. 56.5%; *P* = 0.028) and that most of the gel sponge particles were resorbed within one week of surgery and did not migrate into the surrounding pulmonary arteries and veins [27]. In a retrospective study, Yang et al. [28] reported that the use of gelatin sponge particles to occlude the biopsy tract in patients with emphysema was associated with a lower rate of pneumothorax (20.36% vs. 46.12%, *p* < 0.001) and chest tube placement (3.95% vs. 9.18%, *p* < 0.001) compared with the non-occluded group.

In reviewing the literature, another controversial issue is the site of gelatin sponge injection. Although there are no reports of ectopic embolism after in situ gelatin sponge injection, there have been cases in our center of patients suffering from an extensive cerebral vascular embolism after in situ gelatin sponge injection, which may be caused by damage to the pulmonary veins after puncture and entry of gelatin sponge particles into the systemic circulation. Compared to the peripheral lung area with sparse blood vessels, injection around the tumor with its rich and complex blood vessels still has the potential for ectopic embolism. Therefore, we prefer to inject the gelatin sponge particles after CT confirms that the trocar has been withdrawn 5 mm below the pleura. This is similar to the study by Hadrien Renier et al. [24], and the incidence of post-puncture pneumothorax and safety are identical.

Although most pneumothorax cases are stable and asymptomatic, a specific subset is refractory, necessitating chest tube insertion. However, chest tube placement can lead to related complications and consume additional medical resources that may be initially unnecessary. Therefore, the decision to insert a chest tube must be carefully weighed against potential complications [29, 30]. In our study, the rate of chest tube placement is relatively lower than in previous research [14, 22, 24, 25, 28]. However, interpreting this result demands caution due to variations in technology, equipment across different centers, and the inherent subjectivity of drain placement. Furthermore, it’s worth noting that the difference between the sealing and non-sealing groups did not achieve statistical significance. This may be attributed to the fact that the sealing tract technique is primarily used for patients with high-risk factors, such as emphysema, bullae, long puncture paths, and unavoidable fissure passes, despite using PSM to minimize intra-group differences. Consequently, a comprehensive prospective study is crucial to confirm further the effectiveness of the sealing track method in reducing the necessity for chest tube placement.

Extended and uninterrupted drainage may give rise to complications such as pain, drain blockage, accidental displacement, and, more severely, organ damage, hemothorax, infection, and re-expansion pulmonary edema [31]. Despite the limited number of patients undergoing chest drainage in our study, where no drain blockage was witnessed, we emphasize the importance of the management of drainage occlusion to minimize hospital stays. Blockage of the drainage tube can stem from various factors, including defective drainage tubes like collapsed or twisted ones, drainage tubes protruding from the chest cavity, or incorrect insertion into the chest cavity. Internal chest cavity issues like localized adhesions, encapsulation, and viscous pleural exudate can also contribute. We recommend the following steps to manage drainage blockage: (1) Situation Assessment: Monitor the patient’s condition and drainage flow changes. If a sudden decrease or cessation of flow is noticed, inspect for mechanical issues like twisting, kinking, or external compression. (2) Standard Flushing Procedure: Flushing with saline is advisable if no mechanical faults are found. Typically, gently injecting 10–20 mL of saline using a 20 mL syringe is recommended to restore drainage flow. (3) Drain Replacement: If flushing fails, a medical expert should replace the drain to prevent infections and other complications. (4) Imaging: If internal obstructions like blood clots or tissue blockages are suspected, further assessment with ultrasound or X-ray may be needed. (5) Preventive Actions: Regular monitoring of the drain’s position and functionality and maintaining hygiene and aseptic procedures can significantly reduce blockages. Swift and efficient management is crucial to prevent complications and ensure positive treatment outcomes and patient safety.

Our study has several limitations. First, it is a single-center retrospective study with a small sample size. Although the current study included a large group of patients, the proportion of patients who received gelatin sponge particle embolization was lower than those who did not, which may have affected our comparison. Second, differences in procedural technique, skill, and expertise among interventional radiologists may have influenced the results and outcomes. Thirdly, we did not quantify emphysema and only determined whether emphysema was present before the procedure by CT scan.

## Conclusions

In conclusion, this study shows that using gelatin sponge particles after a CT-guided lung puncture biopsy can effectively prevent the occurrence of pneumothorax. In the future, a large, multi-center, standardized study is needed to verify this result’s universality and long-term effect.

## Data Availability

The datasets used and/or analyzed during the current study are available from the corresponding author upon reasonable request.

## Abbreviations

EMR: Electronic medical records
PACS: Picture archiving and communication systems
PSM: Propensity score matching
PTLB: Percutaneous trans-thoracic lung biopsy
